# Effects of the Topical Administration of Semaglutide on Retinal Neuroinflammation and Vascular Leakage in Experimental Diabetes

**DOI:** 10.3390/biomedicines9080926

**Published:** 2021-07-31

**Authors:** Rafael Simó, Patricia Bogdanov, Hugo Ramos, Jordi Huerta, Olga Simó-Servat, Cristina Hernández

**Affiliations:** 1 Diabetes and Metabolism Research Unit, Vall d’Hebron Research Institute, Universitat Autònoma de Barcelona, 08035 Barcelona, Spain; patricia.bogdanov@vhir.org (P.B.); hugo.ramos@vhir.org (H.R.); jordi.huerta@vhir.org (J.H.); olga.simo@vhir.org (O.S.-S.); cristina.hernandez@vhir.org (C.H.); 2 Centro de Investigación Biomédica en Red de Diabetes y Enfermedades Metabólicas Asociadas (CIBERDEM), Instituto de Salud Carlos III (ISCIII), 28029 Madrid, Spain

**Keywords:** diabetic retinopathy, semaglutide, retina, db/db mouse

## Abstract

Background: An unexpected increase in the rate of severe diabetic retinopathy was observed in the Semaglutide in Subjects with Type 2 Diabetes (SUSTAIN)-6 clinical trial. Although this effect was attributed to a rapid decrease in blood glucose levels, a direct deleterious effect of semaglutide on the retina could not be ruled out. In order to shed light on this issue, we have performed a study aimed at testing the direct effect of semaglutide administered by eye drops on retinal neuroinflammation and microvascular abnormalities using the db/db mouse model. Methods: Eye drops containing semaglutide (0.33 mg/mL; 5 μL once/daily) or vehicle (PBS; 5 μL once daily) were administered for 15 days. Results: We found that semaglutide significantly reduced glial activation, as well as the retinal expression of Nuclear factor kB (NF-κB), proinflammatory cytokines (IL-1β, IL-6, IL-18) and Intercellular Adhesion Molecule (ICAM)-1. In addition, semaglutide prevented the apoptosis of cells from the retinal ganglion layer and activated the protein kinase B (AKT) pathway. Finally, a dramatic decrease in vascular leakage was observed in db/db mice treated with semaglutide. All these findings were observed without any change in blood glucose levels and, therefore, can be directly attributed to semaglutide. Conclusions: These experimental findings point to a beneficial rather than a deleterious effect of semaglutide on the retina of subjects with diabetes.

## 1. Introduction

Neurodegeneration has emerged in recent years as a key element involved in the pathogenesis of early stages of diabetic retinopathy (DR) [1,2]. In fact, the American Diabetes Association (ADA) has defined DR as a highly specific neurovascular complication and, therefore, it is no longer acceptable to consider DR as merely a microvascular complication [3]. However, the Consortium for the Early Treatment of Diabetic Retinopathy (EUROCONDOR) study has shown that a significant proportion of patients present retinal microangiopathic abnormalities without any sign of neurodegeneration [4,5]. This finding suggests that neurodegeneration does not always play a role in the development of DR, and points to drugs that protect both neurons and microvessels as the best candidates for treating early stages of DR. In recent years, experimental evidence has shown that glucagon-like peptide 1 (GLP-1) and its analogues (GLP-1R agonists) are among those drugs with a dual effect: neuroprotective and vasculotropic action [6,7].

However, the results of the Semaglutide in Subjects with Type 2 Diabetes (SUSTAIN) 6 clinical trial, seemed to contravene the beneficial effects of GLP-1R agonists observed in preclinical studies [8]. The SUSTAIN-6 clinical trial showed clear beneficial effects of semaglutide (a GLP-1R agonist) in terms of cardiovascular disease, but an unexpectedly higher rate of advanced DR was observed in patients treated with semaglutide in comparison with those treated with placebo. Among the potential explanations of this finding, the rapid lowering of blood glucose levels was the most plausible. In fact, a post-hoc analysis confirmed this hypothesis and concluded that the magnitude of the reduction of HbA1c and the presence of pre-existing DR were the main factors involved in the risk of DR worsening [9]. Nevertheless, a direct deleterious effect of semaglutide on the retina could not be ruled out.

In order to shed light on this issue, we performed an experimental study aimed at testing the direct effect of semaglutide on early stages of DR using eye drops. The rationale for using eye drops was that we had previously demonstrated that by this route GLP-1R agonists reach the retina without modifying blood glucose levels. Therefore, with this approach, we could examine the direct action of semaglutide independently of its effect on glycemic values. The specific aim was to evaluate the effect of semaglutide administered by eye drops on retinal neurodegeneration, neuroinflammation and early microvascular abnormalities (vascular leakage) in an experimental mouse model.

## 2. Materials and Methods

### 2.1. Animals

A total of 20 male db/db (BKS.Cg-Dock7m +/+ Leprdb/J) mice were purchased from Charles River Laboratories (Calco, Italy). Ten non-diabetic male mice (db/+) matched by age served as a control group. The animals were randomly housed under tight environmental conditions of humidity (60%), temperature (20 °C) and cycles of 12h/12h light/darkness. They had free access to filtered water and “ad libitum” food (ENVIGO Global Diet Complete Feed for Rodents, Mucedola, Milan, Italy).

All animal procedures were conducted in accordance with the Animal Care and Use Committee of VHIR (Vall d’Hebron Research Institute, Passeig de la Vall d’Hebron 119–129, Barcelona, Spain): approval code 75/15, approval date 2 December 2015. In addition the study was performed following the recommendations of the Association for Research in Vision and Ophthalmology Statement for the Use of Animals in Ophthalmic and Vision Research.

### 2.2. Interventional Study

When the diabetic mice were aged 10 weeks, semaglutide eye-drops (*n* = 10) or vehicle eye drops (*n* = 10) were randomly administered. For this purpose, a micropipette (Eppendorf Research^®^ plus pipette 0.5–10 µL Ref. 3123000098; Hamburg, Germany) was used. One drop (5 μL) of semaglutide (0.33 mg/mL), or vehicle (5 μL PBS, pH 7.4) was administered directly onto the superior corneal surface once daily for two weeks in each eye. On the last day, one drop of either semaglutide or vehicle was administered to the eyes 1 h before euthanasia. The evaluation of the results was performed by investigators unaware of the treatment received by the mice.

### 2.3. Neurovascular Damage Assessment

Glial activation was evaluated by Laser Scanning Confocal microscopy using specific antibodies against GFAP (Glial fibrillary acidic protein) as previously reported [7,10]. To evaluate the degree of glial activation, a scoring system based on the extent of GFAP staining previously described was used [11]. The scoring system was as follows: Müller cell end-feet region/ganglion cell layer (GCL) only (score 1); Müller cell end-feet region/GCL plus a few proximal processes (score 2); Müller cell end-feet plus many processes, but not extending to the inner nuclear layer (INL) (score 3); Müller cell end-feet plus processes throughout with some in the outer nuclear layer (ONL) (score 4); Müller cell end-feet plus many dark processes from the GCL to the outer margin of the ONL (score 5). Retinal ganglion cells (RGC) death is characteristic of DR. Thus, the number of cells in the RGC layer was quantified by hematoxylin and eosin (H&E) staining.

Retinal vascular permeability was examined by the assessment of albumin leakage from the blood vessels into the retina using the Evans Blue albumin method (ex vivo). Evans Blue was intraperitoneally injected (E2129 SIGMA, Sant Louis, MO, USA): 17 mg/kg body weight, in a concentration of 5 mg/mL dissolved in PBS pH 7.4. Immediately after injection, the animals visibly turned blue, confirming dye uptake and distribution. After 120 min, the mice were euthanized by cervical dislocation and the eyes were enucleated. Flat-mounted slides were obtained, and coverslipped with a drop of mounting medium Prolong Gold antifade reagent (Invitrogen, Thermo Fisher Scientific, Waltham, MA, USA). Digital images from different random fields of all the retinas were acquired using a confocal laser scanning microscope (FV1000; Olympus, Hamburg, Germany) at 60× using the 561-nm laser line, and each image was recorded with identical beam intensity at a size of 1024 pixels × 1024 pixels. For analysis of the albumin-bound Evans Blue, Z-stack retinal images (step size 1.16 μm) of different regions of the vascular tree were acquired. For quantitative analysis of the albumin-bound Evans Blue, the number of extravasations per field of 60× was counted.

### 2.4. Mechanism of Action

Pro-survival signaling (AKT/p-AKT) and inflammation (NFκB, proinflammatory cytokines and ICAM-1) were analyzed by RT-PCR, Western blot, and immunohistochemistry.

### 2.5. RNA Isolation and Quantitative RT-PCR assay

Total RNA from mice was extracted using Trizol^®^ reagent (Invitrogen, Madrid, Spain) according to the manufacturer’s protocol. Then, RNA samples were treated with DNase (Qiagen, Madrid, Spain) to remove genomic contamination and purified on an RNeasy MinElute column (Qiagen, Madrid, Spain). RNA quantity was measured on a Nanodrop spectrophotometer, and integrity was determined on an Agilent 2100 Bioanalyzer. The single-strand cDNA was synthesized as described in Prime Script^®^ RT Master Mix kits. Real-time RT-PCR was performed using SYBR Green PCR Master Mix (Applied Biosystems, Warrington, UK) using the 7.900 HT Sequence Detection System in 384-well optical plates with specific primers displayed in Table 1.

Each sample was assayed in triplicate. Applied Biosystems 7900HT Fast Real-Time PCR System (ABI SDS 2.0 RQ, Applied Biosystems, Waltham, MA, USA) software and the 2−ΔΔCt analysis method were used for relative quantification (R.Q.) calculation with Beta-2-Microglobin (*B2M*) as endogenous control.

### 2.6. Western Blotting

A lysis buffer was prepared (RIPA buffer: phenylmethanesulfonylfluoride [PMSF], 1 mM; Na3VO4, 2 mM; NaF, 100 mM. Sigma, St Louis, MO, USA) with a 1× protease inhibitor cocktail (Sigma, St Louis, MO, USA). Then, proteins were extracted from the neuroretinas in 80 μL of the aforementioned buffer. A total of 25 μg protein was resolved by 10% SDS-PAGE and transferred to a PVDF membrane (Bio-Rad Laboratories, Madrid, Spain). The primary antibodies (Table 2) were incubated overnight at 4 °C.

The following day, the suitable HRP secondary antibody anti-rabbit or anti-mouse (Dako Agilent, Santa Clara, CA, USA) was incubated for 1 h at room temperature. Cyclophilin (CYP) and vinculin were used to normalize protein levels. Densitometric analysis of the western blot bands was performed with Image J software (National Institutes of Health, Bethesda, MD, USA).

### 2.7. Immunohistochemical Analysis

Firstly, paraffined sections were deparaffinized in xylene (VWR, Barcelona, Spain) and rehydrated in ethanol (Sigma, St Louis, MO, USA). Sections were fixed in acid methanol (−20 °C) for 1 min and washed with 0.01 M 4 phosphate-buffered saline (PBS; Biowest, Labclinics, Barcelona, Spain) at pH 7.4. Then, sections were incubated in blocking solution (3% BSA, Tween 0.05% PBS; Sigma Aldrich, St Louis, MO, USA) for 1 h at room temperature and afterwards, they were incubated overnight at 4 °C with a specific primary antibody (anti-GFAP rabbit monoclonal; 1:500; ab7260; Abcam, Cambridge, UK). The following day, after washing, sections were incubated with a fluorescent ALEXA 488 as a secondary antibody (anti-rabbit or anti-mouse) (Life Technologies S.A, Madrid, Spain) in blocking solution (Protein Block Serum-Free Ready-To-Use DAKO Agilent X0909, Agilent Technologies, Inc., Santa Clara, CA, USA) for 1 h and washed again. Nuclei were counterstained using Hoechst 33,342 (2′-[4-ethoxyphenyl]-5-[4-methyl-1-piperazinyl]-2,5′-bi-1H-benzimidazole trihydrochloride trihydrate), an organic compound that emits blue fluorescence when bound to DNA (Thermo Fisher Scientific, OR, USA). Finally, samples were mounted in Mounting Medium Fluorescence (Prolong, Invitrogen, Thermo Fisher Scientific, OR, USA) with a coverslip. Images were acquired with a confocal laser scanning microscope (FV1000; Olympus, Hamburg, Germany) at a resolution of 1024 × 1024 pixels. Five fields (three corresponding to the central and two to the peripheral retina) from each section were analyzed using ImageJ software (National Institutes of Health, Bethesda, MD, USA).

### 2.8. Statistical Analysis

The results are expressed as the mean ± Standard Error (SE). Statistical comparisons were performed with a Student’s unpaired test. When multiple comparisons were performed, one-way ANOVA followed by the Bonferroni test was used. For statistical purposes, the GFAP extent score was categorized as “normal” (scores 1 and 2) and “pathological” (scores 3, 4 and 5), and for comparisons between groups the Fisher’s exact test was used. Levels of statistical significance were set at *p* < 0.05.

## 3. Results

**Figure 1:**
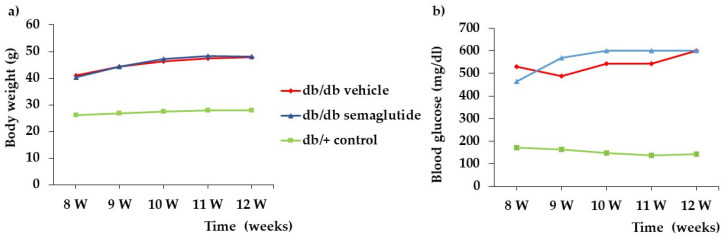
Evolution of blood glucose (**a**) and body weight (**b**) in the experimental groups.

We did not find any difference in blood glucose concentrations and body weight during the study between db/db mice treated with semaglutide and db/db mice treated with vehicle (Figure 1).

### 3.1. Effect of Semaglutide on Neurovascular Unit

**Figure 2:**
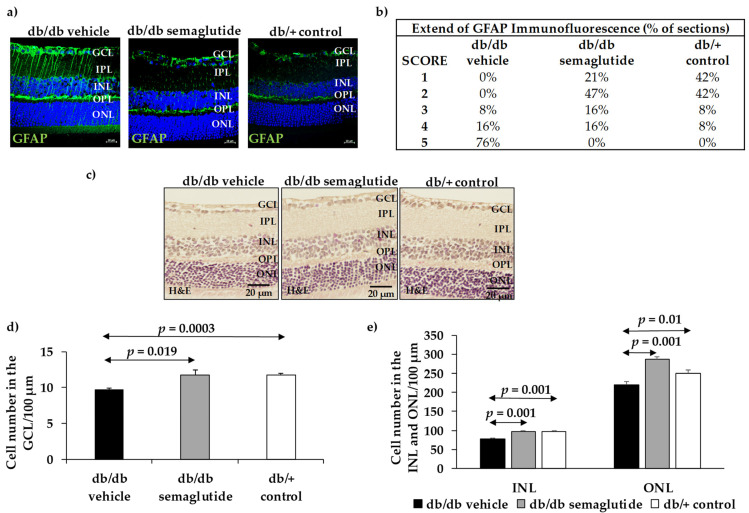
Effect of semaglutide eye drops on glial activation and apoptosis. (**a**) Comparison of GFAP immunoreactivity (green) in the retina among representative samples from a diabetic mouse treated with vehicle, a diabetic mouse treated with semaglutide, and a non-diabetic mouse. Nuclei were labeled with Hoechst (blue). ONL: outer nuclear layer; OPL: outer plexiform layer; INL: inner nuclear layer; IPL: inner plexiform layer; GCL: ganglion cell layer. Scale bars, 20 µm. (**b**) Quantification of glial activation based on the extent of GFAP staining. (**c**) Comparison of hematoxylin/eosin (HE) retinas among representative samples from diabetic mice treated with vehicle, semaglutide and from a non-diabetic mouse. (**d**) Quantification of the number of cells in GCL. (**e**) Quantification of the number of cells in the INL and ONL.

As expected, in non-diabetic control mice (db/+) GFAP expression was mainly confined to the retinal ganglion cell layer (GCL) (Figure 2a,b). The diabetic mice (db/db) treated with vehicle presented significantly higher GFAP expression than non-diabetic mice matched by age (Fisher’s exact test: *p* < 0.001). It should be noted that no diabetic mice treated with the vehicle presented a GFAP score <3. The administration of semaglutide resulted in a significant decrease in reactive gliosis, the GFAP score being <3 in 68% of cases (Figure 2b) (Fisher’s exact test: *p* < 0.001 between diabetic mice treated with vehicle and diabetic mice treated with semaglutide).

The number of cells of GCL was significantly lower in db/db mice treated with vehicle than in non-diabetic mice (9.74 ± 0.32 vs. 11.71 ± 0.51; *p* < 0.01). Topical treatment with semaglutide led to a significant decrease in apoptosis in the GCL, the INL and the ONL in diabetic mice (Figure 2c–e).

**Figure 3:**
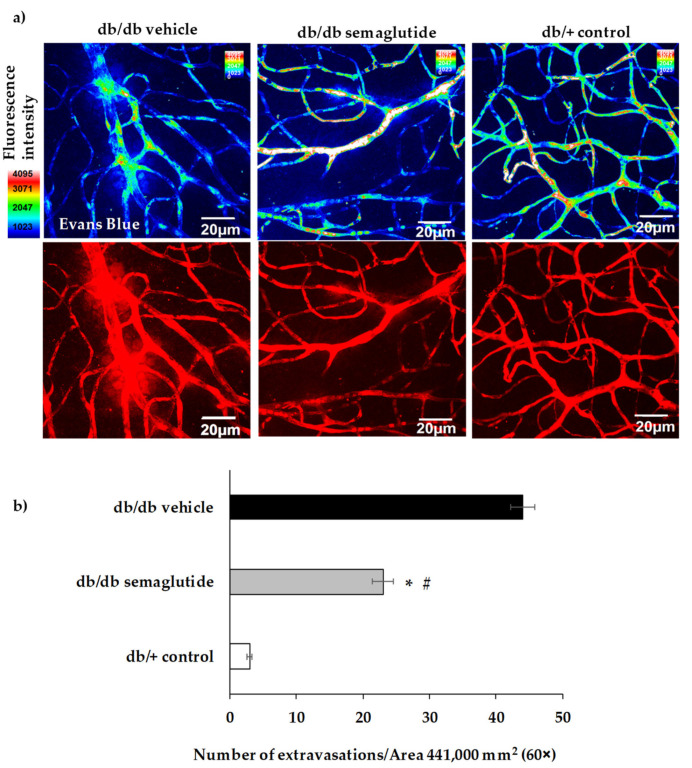
Effect of semaglutide eye drops on vascular leakage. (**a**) Confocal immunofluorescence images of vascular permeability assessed by Evans Blue dye leakage in retinal whole mounts. Spec3, fluorescent spectral signature 3. FI: fluorescence intensity. Scale bars, 20 μm. (**b**) For quantification, the number of extravasations per field of 60× retina was counted. * *p* < 0.05 in comparison with vehicle. # *p* < 0.05 in comparison with control.

The number of vascular extravasations was significantly reduced in diabetic mice treated with semaglutide in comparison with diabetic mice treated with placebo (Figure 3).

### 3.2. Mechanisms of Action

**Figure 4:**
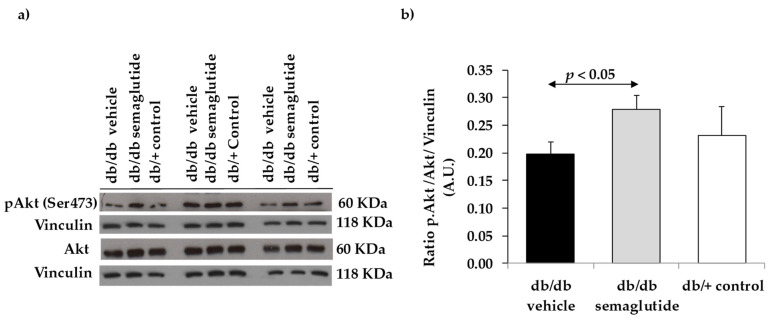
Effect of semaglutide eye drops on cell survival. (**a**) Western blotting bands of phospho-Akt/Akt. (**b**) Densitometric analyses in mouse retinas of db/db mice treated with vehicle (black bars), db/db mice treated with semaglutide eye drops (grey bars) and non-diabetic mice (white bars).

Semaglutide leads to the activation of a downstream specific prosurvival pathway, the adenylyl cyclase and phosphatidylinositol 3-kinase/Akt pathway. We found a significant increase in ratio pAkt/Akt in the retinas of diabetic mice treated with semaglutide, suggesting that the activation of GLP-1R was produced (Figure 4).

**Figure 5:**
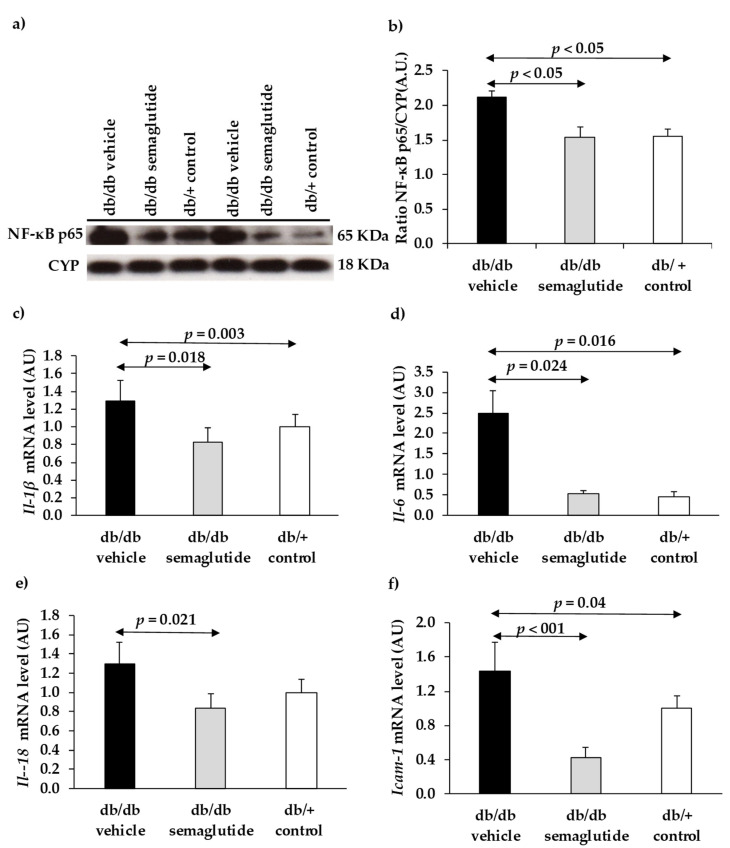
Effect of semaglutide eye drops on retinal inflammation. (**a**) Western blotting bands of NF-κB (p65). (**b**) Densitometric analyses in mouse retinas in db/db mice treated with vehicle (black bar), db/db mice treated with semaglutide (grey bar) and non-diabetic mice (white bar). (**c**–**f**) Real-time quantitative reverse transcription polymerase chain reaction (RT-PCR) analysis of IL-1β (**c**), IL-6 (**d**), IL-18 (**e**), and ICAM-1 (**f**).

We found that topical administration of semaglutide produced a down-regulation of NF-κB in the retina. Thus, diabetic mice treated with semaglutide exhibited a significantly lower content of active NF-κB (p65) protein than diabetic mice treated with vehicle and similar to that of non-diabetic mice (Figure 5a,b). Retinas from diabetic mice treated with vehicle showed a significant upregulation (mRNA) of several proinflammatory cytokines such as Il-1β, Il-6 and Il-18, as well as Icam-1 (Figure 5c–f). Treatment with semaglutide abrogated this upregulation, leading to levels similar to the non-diabetic control group.

## 4. Discussion

Our experimental findings suggest that the topical administration of semaglutide is effective in preventing retinal neurodegeneration, neuroinflammation and vascular leakage induced by diabetes. Therefore, rather than a deleterious effect, semaglutide exhibits beneficial results as has been reported with other GLP-1R agonists using experimental models [6,7,12,13].

It has been reported that intravitreal injection of exendin-4 prevents functional and morphologic abnormalities related to neurodegeneration in rats with streptozotocin-induced diabetes [12,13] and in Goto-Kakizaki rats [6]. We observed similar effects using eye-drops containing native GLP-1 or its analogues (liraglutide, exenatide and lixi-senatide), as well as with the subcutaneous administration of liraglutide [7]. In addition, these neuroprotective actions were accompanied by the prevention or even the regression of diabetes-induced vascular leakage [6,7,14]. Taken together, these findings suggest that GLP-1R agonists have a dual effect (neuroprotective and vasculotropic) which ameliorates the diabetes-induced impairment of the neurovascular unit [15].

The absence of a significant modification in blood glucose levels after topical (eye drops) administration of semaglutide has the advantage that the confounding factor of the concomitant improvement of metabolic control that occurs after initiating the subcutaneous administration of semaglutide is avoided. It should be noted that, in humans, long-term metabolic control is associated with a reduction in the incidence and progression of DR, but also that a rapid improvement of blood glucose levels has been associated with an early worsening of DR. Therefore, it is very difficult to know whether the effect of GLP-1R agonists or other antidiabetic agents on the development and progression of DR is merely due to their efficacy in lowering blood glucose levels or whether they have any direct effect per se. In this regard, the design of our study permitted us to test the direct effect of semaglutide on the diabetic retina independently of its antidiabetic action. The effects observed in the retina were unrelated to systemic effects and should be attributed to its direct action at the retinal level. In addition, recent pharmacokinetic data in mice suggest that semaglutide is unable to cross the blood-brain barrier (BBB) [16]. Since the transport and permeation characteristics are similar between the BBB and the blood-retinal barrier (BRB), it seems unlikely that semaglutide when administered by systemic route could reach the retina, at least at early stages of DR where a significant disruption of the BRB does not exist. By contrast, in eye drops, semaglutide can reach the retina without crossing the BRB.

Regarding the mechanisms of action, we provide evidence that topical administration of semaglutide leads to a powerful anti-inflammatory action by downregulating the expression of NFKB, proinflammatory cytokines (IL-1β, IL-6, IL-18) and ICAM-1. In addition, semaglutide prevents the apoptosis of neuroretinal cells by activating the Akt pathway, which is essential for neuron survival. Besides preventing diabetes-induced neurodegeneration, topical administration of semaglutide inhibits the disruption of the blood-retinal barrier and the subsequent vascular leakage, thus confirming the dual effect of GLP-1R agonists.

Our findings argue against a potential toxic effect of semaglutide at the retinal level. However, it might be hypothesized that the worsening of DR observed in the SUSTAIN-6 study could be partly accounted for by the inclusion of patients with advanced non-proliferative DR or proliferative DR in whom semaglutide could have triggered neovascularization. At this point it should be noted that GLP-1R agonists, administered by either eye drops or subcutaneously, downregulate the retinal expression of VEGF in db/db mice [7], thus making VEGF mediated angiogenesis very unlikely. In addition, there is no information regarding any potential in vitro or in vivo angiogenic effect of GLP-1R agonists in the retina. Furthermore, a careful review of data reported in the SUSTAIN-6 study shows that in the first year there were 21 (58%) “events” (advanced DR) in the semaglutide arm vs. 14 (48%) in the placebo arm, whereas in the second year 21 (42%) “events” in the semaglutide arm vs. 15 (51%) in the placebo arm were reported. This information revealed that semaglutide does not have any toxic or angiogenic effect because if this was the case a worsening rather than an improvement in advanced DR rate would be expected.

It could be argued that our experiment was too short for detecting any toxicity related to semaglutide. However, it should be noted that in the SUSTAIN-6 study there were 12 “events” in the semaglutide arm vs. only one “event” in the placebo arm at week 16. Therefore, the planned duration of our study seems appropriate. To the best of our knowledge there is no drug that could develop similar lesions to those that occur in advanced DR in this short period, thus suggesting a randomization bias in terms of the degree of DR at study entry. In this regard, SUSTAIN 6 was designed for monitoring cardiovascular disease but not DR. Taken together, the higher number of patients with advanced DR included at study entry in the semaglutide arm and the rapid effect in lowering blood glucose levels would seem the most plausible explanation of the results obtained in SUSTAIN-6 regarding DR.

In conclusion, our experimental findings point to a beneficial rather than a deleterious effect of semaglutide on the retina of subjects with diabetes. A large clinical trial (FOCUS study) with four years of follow-up aimed at answering this question is ongoing. Meanwhile, as recommended for all the antidiabetic drugs, it seems reasonable to use semaglutide in the type 2 diabetic population with mild-moderate DR, but in those patients with severe non-proliferative or proliferative DR particular care in avoiding a rapid reduction of blood glucose levels seems warranted.

## Figures and Tables

**Table 1:** Primers used for RT- PCR.

Gen Symbol	Sequence Primer
*Il 1 β* Forward (5′-3′)	5′-GCAACTGTTCCTGAACTCAACT-3′
*Il 1 β* Reverse (5′-3′)	5′-ATCTTTTGGGGTCCGTCAACT-3′
*Il 6* Forward (5′-3′)	5′-TAGTCCTTCCTACCCCAATTTCC-3′
*Il 6* Reverse (5′-3′)	5′-TTGGTCCTTAGCCACTCCTTC-3′
*Il 18* Forward (5′-3′)	5′-AGCAGTCCCAACTAAGCAGTA-3′
*Il 18* Reverse (5′-3′)	5′- CAGCCAGTAGAGGATGCTGA-3′
*Icam-1* Forward (5′-3′)	5′- GGCATTGTTCTCTAATGTCTCCG-3′
*Icam-1* Reverse (5′-3′)	5′- TGTCGAGCTTTGGGATGGTAG-3′
*B2M* Forward (5′-3′)	5′- GGCCCATCTTGCATTCTAGGG-3′
*B2M* Reverse (5′-3′)	5′- CAGGCAACGGCTCTATATTGAAG-3′

**Table 2:** Primary antibodies and specific dilutions used in Western Blot analysis.

Antibody	Description
NF-κB (p65)	1:1000; sc-8008; Santa Cruz Biotechnology Inc, Dallas, Texas, USA
p-AKT	1:1000; #2965; Cell Signaling, Leiden, The Netherlands
AKT	1:1000; #9272; Cell Signaling, Leiden, The Netherlands
Cyclophilin A	1:10000; BML-SA296; Enzo, NY, USA
Vinculin	1:100,000; sc-73614; Santa Cruz Biotechnology Inc, Dallas, TX, USA

## Data Availability

The data presented in this study are available on request from the corresponding author.
